# Genomic profiling of soil nitrifying microorganisms enriched on floating membrane filter

**DOI:** 10.71150/jm.2502002

**Published:** 2025-04-29

**Authors:** Christiana Abiola, Joo-Han Gwak, Ui-Ju Lee, Aderonke Odunayo Adigun, Sung-Keun Rhee

**Affiliations:** Department of Biological Sciences and Biotechnology, Chungbuk National University, Cheongju 28644, Republic of Korea

**Keywords:** floating membrane filter cultivation, metagenomic sequencing, soil nitrifiers, ammonia-oxidizing archaea, *Nitrososphaeraceae*

## Abstract

Recently, floating membrane filter cultivation was adopted to simulate solid surface and enrich surface-adapted soil ammonia-oxidizing archaea (AOA) communities from agricultural soil, as opposed to the conventional liquid medium. Here, we conducted metagenomic sequencing to recover nitrifier bins from the floating membrane filter cultures and reveal their genomic properties. Phylogenomic analysis showed that AOA bins recovered from this study, designated FF_bin01 and FF_bin02, are affiliated with the *Nitrososphaeraceae* family, while the third bin, FF_bin03, is a nitrite-oxidizing bacterium affiliated with the *Nitrospiraceae* family. Based on the ANI/AAI analysis, FF_bin01 and FF_bin02 are identified as novel species within the genera “*Candidatus* Nitrosocosmicus” and *Nitrososphaera*, respectively, while FF_bin03 represents a novel species within the genus *Nitrospira*. The pan and core genome analysis for the 29 AOA genomes considered in this study revealed 5,784 orthologous clusters, out of which 653 were core orthologous clusters. Additionally, 90 unique orthologous clusters were conserved among the *Nitrososphaeraceae* family, suggesting their potential role in enhancing culturability and adaptation to diverse environmental conditions. Intriguingly, FF_bin01 and FF_bin02 harbor a gene encoding manganese catalase and FF_bin03 also possesses a heme catalase gene, which might enhance their growth on the floating membrane filter. Overall, the floating membrane filter cultivation has proven to be a promising approach for isolating distinct soil AOA, and further modifications to this technique could stimulate the growth of a broader range of uncultivated nitrifiers from diverse soil environments.

## Introduction

The oxidation of ammonia (NH_3_) to nitrite (NO_2_^-^), the first and rate-limiting step in nitrification, is generally carried out by ammonia-oxidizing archaea (AOA) and ammonia-oxidizing bacteria (AOB) (Beeckman et al., 2018). Meanwhile, NO_2_^-^ is subsequently oxidized to nitrate (NO_3_^-^) by nitrite-oxidizing bacteria (Starkenburg et al., 2014). Recently, complete ammonia oxidizers (comammox), capable of oxidizing ammonia to nitrate, were discovered within the NOB genus *Nitrospira* (Daims et al., 2015; Van Kessel et al., 2015).

Phylogenetically, AOA are members of the class *Nitrososphaeria* within the phylum *Nitrososphaerota*, formerly *Thaumarchaeota* (Oren, 2024). The class *Nitrososphaeria* includes four major lineages: “*Candidatus* Nitrosocaldales” (thermophilic AOA clade), *Nitrosopumilales* (formally Group I.1a), “*Ca*. Nitrosotaleales” (Group I.1a-associated), and *Nitrososphaerales* (formally Group I.1b) (Sheridan et al., 2020). Recently, Zheng et al. (2024) proposed a fifth novel order-level lineage of AOA, designated “*Ca*. Nitrosomirales”, which is prevalent in marine and terrestrial environments. In soil ecosystems, the *Nitrososphaeraceae* family, belonging to the *Nitrososphaerales* lineage, frequently dominates among AOA populations, with some cultivated representatives within the genera “*Ca*. Nitrosocosmicus” and *Nitrososphaera* and various subclusters that have not been cultured (Bates et al., 2011; Ochsenreiter et al., 2003).

Previous research on soil AOA of the *Nitrososphaerales* indicated they exhibit a higher genetic capability for producing extracellular polymeric substances (EPS), which are essential for biofilm or microcolony formation, compared to soil AOA of the *Nitrosopumilales* (Kerou et al., 2016). Abiola et al. (2024) recently reported that *Nitrososphaera viennensis* EN76, a member of the *Nitrososphaeraceae* family, formed microcolonies on floating membrane filters, whereas “*Nitrosotenuis chungbukensis*” MY2 of the family *Nitrosopumilaceae* couldn’t thrive on the floating membrane filter. Transcriptomic analysis of *N. viennensis* EN76 floating filter-grown cells revealed upregulation of unique sets of genes for cell wall and EPS biosynthesis, and defense against H_2_O_2_-induced oxidative stress. Although there is a recent report of two AOA strains forming visible colonies on a solid-liquid medium (Klein et al., 2022), the floating membrane filter cultivation technique might be promising, as it effectively enriched distinct soil AOA communities dominated by the genus “*Ca*. Nitrosocosmicus” within the *Nitrososphaeraceae* family, as opposed to the conventional liquid media (Abiola et al., 2024). Herein, we conducted metagenomic sequencing to recover nitrifier bins from our floating membrane filter cultures and reveal their genomic properties. In addition, we performed comparative genomic analysis using 27 AOA reference genomes and the AOA bins recovered in this study.

## Materials and Methods

### Cultivation on floating membrane filter

Floating membrane filter cultivation technique was adopted to enrich surface-adapted AOA from agricultural soil. Artificial freshwater medium (AFM) supplemented with calcium carbonate particles (4 g/L of medium) was used as the growth medium for cultivation. To specifically target AOA communities, allylthiourea at a concentration of 50 µM, which effectively inhibits bacterial ammonia-oxidizers, was added to the growth medium as previously described by Abiola et al. (2024). Briefly, the medium was supplemented with 1 mM ammonium chloride (NH_4_Cl) as the sole energy source. Pre-rinsed sterile nucleopore polycarbonate (PC) track-etched filters (Cytiva, USA) with a diameter of 47 mm and a pore size of 0.1 µm were used for the experiment. The filter was mounted on the autoclaved filter holders (Thermo Fisher Scientific, USA). Afterward, one milliliter (1 ml) of 10^-2^-fold diluted soil suspension was mixed with 4 ml AFM and vacuum-filtered at 13 mbar. Then, the filter was aseptically placed on top of 10 ml medium in a sterile polystyrene petri dish (60 × 15 mm) using sterile tweezers. The petri dishes were placed in a sealed container with ambient air and incubated in the dark at 30°C without shaking. Nitrification activity was monitored by subsampling from the liquid medium beneath the filter and measuring NO_2_^-^ and NO_3_^-^ accumulation. The concentrations of NO_2_^-^ and NO_3_^-^ were quantified colorimetrically using the Griess and VCl3/Griess reagents, respectively (Kits et al., 2017). Similarly, ammonia concentration was determined colorimetrically using the method described by Solorzano (1969) and Jung et al. (2011).

After ca. 0.8 mM of ammonia was oxidized by the floating membrane filter cultures, the filter containing the grown cells was submerged in 10 ml of AFM in the petri dish and scraped with a cell scraper. Ten percent (10%) of the resulting cell suspension was used to repeat the filtration procedure as previously described. Ammonia oxidation was continuously monitored, and subsequent transfers were made at the mid-exponential phase (biweekly) for up to 11 months. Afterward, the cells on the filters were harvested for metagenomic sequencing.

### Metagenome sequencing

DNA was extracted by using the modified hexadecyltrimethylammonium bromide (CTAB)-based protocol (Hurt et al., 2001). Long-read sequencing libraries were constructed using the Native Barcoding Kit 24 V14 (SQK-NBD114.24), and sequencing was performed on the MinION platform (Oxford Nanopore Technology) utilizing an R10.4.1 flow cell (FLO-MIN114). Base-calling was carried out with Dorado (v0.8.0) (https://github.com/nanoporetech/dorado/), integrated within MinKNOW software (v23.04.6) (https://github.com/nanoporetech/minknow_api). A total of 1,361,962,030 bp were produced from 1,843,806 reads (maximum read length: 221,746 bp), with a median quality score of 30.09, corresponding to an accuracy of approximately 99.9%.

### Genome scaffold assembly, binning, and annotation

Metagenomic assembly was conducted using metaFlye (v2.9.2) (Kolmogorov et al., 2020), followed by error correction with Medaka (v1.9.1) (https://github.com/nanoporetech/medaka). Contig coverage was determined by mapping raw reads with Minimap2 (v2.26) (Li, 2018), and the resulting BAM files were sorted using SAMtools (v1.3.1) (Li et al., 2009). Sequencing depth was calculated with the jgi_summarize_bam_contig_depths script. Binning was performed through differential coverage binning using the mmgenome2 R package (https://github.com/KasperSkytte/mmgenome2) (Albertsen et al., 2013).

Taxonomic annotation of the bins was conducted with Prokka (v1.14.5) (Seemann, 2014). Homologous genes related to metabolic pathways were identified through BLAST searches against non-redundant protein sequences in the KEGG (Kanehisa et al., 2016) and UniRef90 (Suzek et al., 2007) databases. Functional assignment of the predicted genes was refined using a suite of public databases, including InterPro (Paysan-Lafosse et al., 2023), Gene Ontology (Ashburner et al., 2000; Consortium, 2023), Pfam (Mistry et al., 2021), the Conserved Domains Database (Wang et al., 2023), TIGRFAM (Selengut et al., 2007), and EggNOG (Hernández-Plaza et al., 2023). Signal peptide prediction and transmembrane helix identification were performed using the web-based tools SignalP (v5.0) (Almagro Armenteros et al., 2019) and TMHMM (v2.0) (Krogh et al., 2001) with default settings.

### Phylogenomic analysis

Phylogenomic analysis of the nitrifier bins recovered in this study was performed using the Anvi'o phylogenomic workflow (Eren et al., 2021). Briefly, a concatenated alignment of 76 single-copy core archaeal genes and 71 single-copy bacterial genes from selected reference genomes obtained from the NCBI databases, was used to construct the phylogenomic tree. The constructed tree was visualized using iTOL (v6.0) (Letunic and Bork, 2024).

### Comparative genomic analysis

Bin quality was evaluated using CheckM v.1.0.7 (Parks et al., 2015). AOA genome similarity was determined using genome-relatedness parameters, including average nucleotide identity (ANI) and average amino acid identity (AAI). The ANI values between the pair of complete genomes were calculated by using the OrthoANI program (Yoon et al., 2017), and the AAI values were calculated by CompareM (v0.1.2) (https://github.com/dparks1134/CompareM). The heatmaps were generated according to the ANI and AAI values by using R (v4.3.1) (R Core Team, 2021).

A comparative genomic analysis was conducted with an additional 27 AOA reference genomes, each with estimated genome completeness ≥ 90% and contamination ≤ 10%. For all the 29 genomes analyzed, we identified single-copy orthologous sequences using OrthoFinder (v2.5.5) (Emms and Kelly, 2019). To define the pangenome, all the orthologous clusters were further analysed in R (v4.3.1). A concentric circle plot was created using ggplot2 (Wickham, 2016). Additionally, the OrthoVenn3 web server (Sun et al., 2023) was employed with default parameters (E-value 0.01; inflation value 1.50) to compare orthologous clusters among the closest enriched genomes within the *Nitrososphaeraceae* family.

## Results and Discussion

**Fig. 1.**
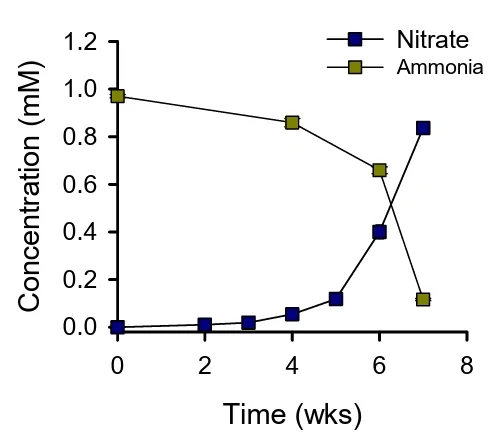
Ammonia oxidation by the floating membrane filter cultures. The concentrations of ammonia and nitrate are indicated. All experiments were performed in triplicates. Data are presented as mean ± standard deviation (SD) (*n* = 3), and the error bars are hidden when they are smaller than the width of the symbols.

Nitrification activity of the enrichment cultures was ascertained by the accumulation of NO_3_^-^ in the media beneath the filter. The initial culture took 7 weeks to oxidize ca. 0.8 mM of ammonia (Fig. 1). Subsequent transfers using 10% of the biomass scraped from the filter required 2 weeks to oxidize ca. 0.7 mM of ammonia. After several rounds of transfer for a period of 11 months, the cells on the filters were harvested for metagenomic sequencing. Three bins were recovered from the floating membrane filter culture. Two of these bins, designated FF_bin01 and FF_bin02, are ammonia-oxidizing archaea (AOA) affiliated with the *Nitrososphaeraceae* family. The third bin, FF_bin03, is a nitrite-oxidizing bacterium belonging to the *Nitrospiraceae* family. The family *Nitrospiraceae* plays a crucial role in soil nitrification and are often found in close association with AOA or AOB (Daims and Wagner, 2018).

The genome of FF_bin01 had the highest scaffold coverage of 119× (Table S1), with genome completeness of 95.15% and contamination of 2.91%. The genome has an average GC content of 34.28% with 3,248 protein-coding genes. Similarly, FF_bin02 had genome completeness of 97.09% with scaffold coverage of 81× and contamination of 0.97%, with an average GC content of 55.79% and 2,403 protein-coding genes. Further, the draft genomes of FF_bin03 had genome completeness of 87.67% and contamination of 14.26%, with a scaffold coverage of 29×. This bin was not identified as *Nitrospira* comammox. An overview of all the bins recovered in this study is provided in Table S1.

**Fig. 2.**
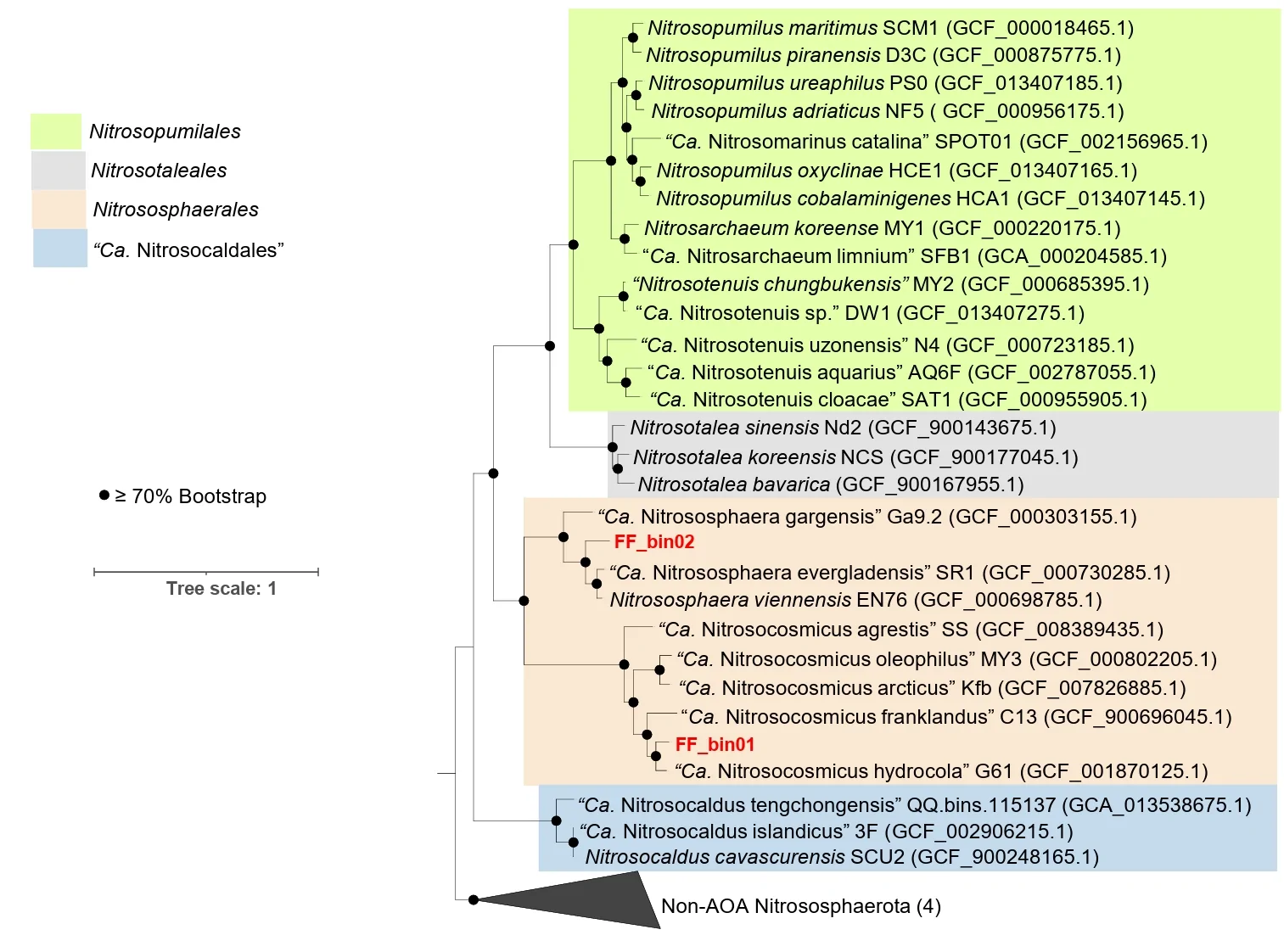
A maximum likelihood phylogenomic tree of recovered AOA bins and reference genomes. The tree was inferred based on the concatenated alignment of 76 single-copy core archaeal genes; the non-ammonia-oxidizing archaea (Non-AOA) *Nitrososphaerota* were used as an outgroup. Bootstrap values ≥ 70% based on 1,000 replications are indicated. The scale bar represents a 1 change per nucleotide position. The two AOA bins recovered in this study are highlighted in red.

The genomic features of the AOA bins, including genome size, GC content, predicted protein-coding genes, and various growth features compared with other AOA strains, are described in Table 1. For comparative analysis, we only considered reference AOA genomes with isolated or highly enriched laboratory cultures. Based on the phylogenomic analysis, FF_bin01 genome formed a highly supported cluster with the genus “*Ca*. Nitrosocosmicus”, whereas FF_bin02 genome formed a cluster with the genus *Nitrososphaera* (Fig. 2). The ANI and AAI values between FF_bin01 and other “*Ca*. Nitrosocosmicus” genomes, as well as between FF_bin02 and other *Nitrososphaera* genomes, were well below the thresholds recommended for species delineation (Fig. S1A and S1B) (Ciufo et al., 2018; Richter and Rosselló-Móra, 2009) suggesting FF_bin01 and FF_bin02 represent novel species within the genera “*Ca*. Nitrosocosmicus” and *Nitrososphaera*, respectively.

**Fig. 3.**
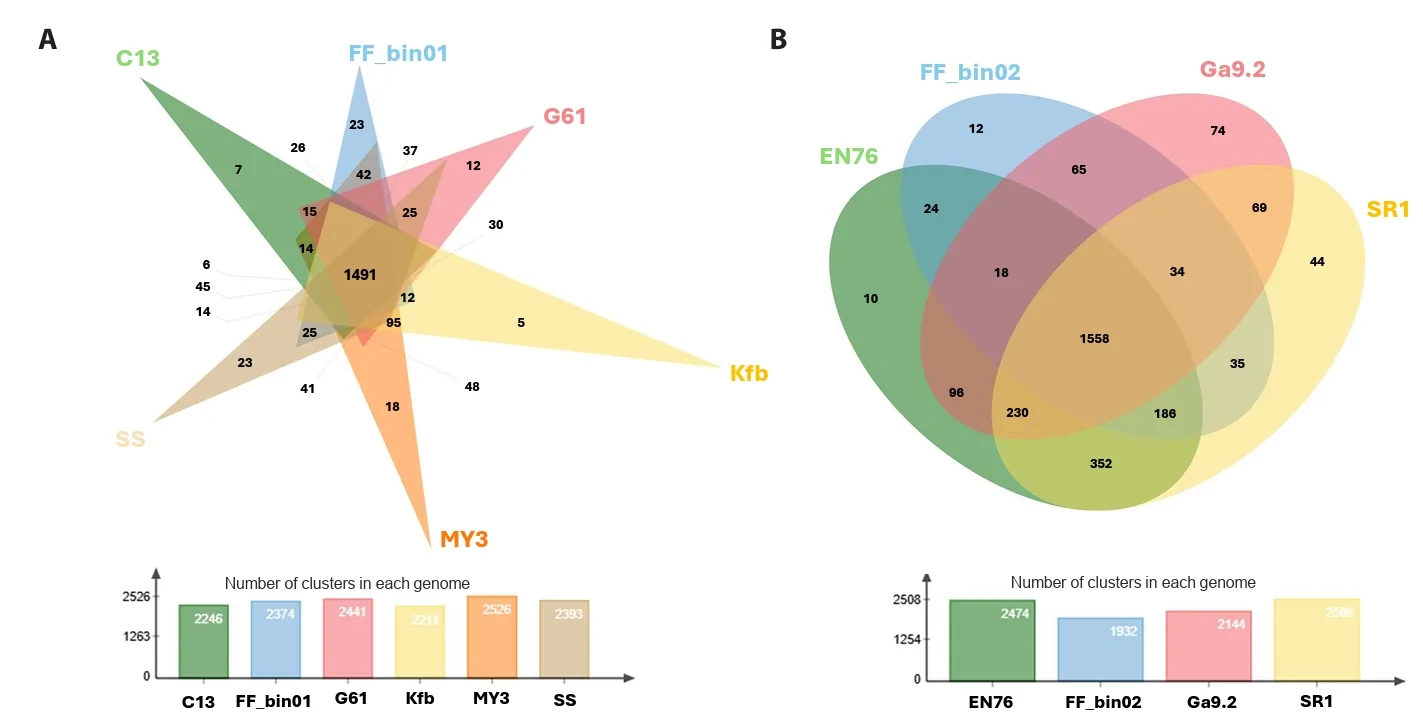
Orthologous cluster identification and comparative analysis. A Venn diagram displaying the number of unique and shared clusters among (A) the genus “*Ca*. Nitrosocosmicus” and (B) the genus *Nitrososphaera*, complemented by a bar chart showing the number of clusters in each genome. Each genome was represented with its strain ID, “*Ca*. Nitrosocosmicus franklandus” C13 = C13; “*Ca*. Nitrosocosmicus agrestis” SS = SS; “*Ca*. Nitrosocosmicus hydrocola” G61 = G61; “*Ca*. Nitrosocosmicus arcticus” Kfb = Kfb; “*Nitrosocosmicus oleophilus*” MY3 = MY3; strain FF_bin01, *Nitrososphaera viennensis* EN76 = EN76, “*Ca*. Nitrososphaera evergladensis” SR1 = SR1; “*Ca*. Nitrososphaera gargensis” Ga9.2 = Ga9.2; strain FF_bin02.

The pan and core genomic analysis of 27 AOA reference genomes with our AOA bins revealed 5,784 orthologous clusters, out of which 653 were core orthologous clusters. A total of 1,645 and 3,486 were identified as cloud and shell orthologous clusters, respectively (Fig. S2A and S2B). It is worth pointing out that we only considered the isolated or enriched laboratory AOA cultures. Core orthologous clusters simply mean groups of orthologs (or paralogs) identified in all the AOA genomes. They are integral to essential biological processes, including ammonia oxidation and carbon fixation via the 3-hydroxypropionate/4-hydroxybutyrate pathway (Könneke et al., 2014). The shell orthologous clusters were found in at least three of the AOA genomes, while cloud orthologous clusters were present in not more than two. We further showed the shared and conserved orthologous clusters among the “*Ca*. Nitrosocosmicus” and *Nitrososphaera* (Fig. 3A and 3B).

Ninety (90) unique conserved orthologous clusters among the *Nitrososphaeraceae* family (Supplementary Table) encompass proteins associated with signal transduction, glycosyl transferase (family group II), adenylate cyclase (class II and III), flavodiiron proteins, thioredoxin-like proteins, extra copies of genes involved in electron transfer (including genes encoding the blue copper domain-containing proteins), transcriptional factors/regulators, and some proteins of unknown functions. It is worth noting that, to date, only a few “*Ca*. Nitrosocosmicus” and *Nitrososphaera* species have been isolated or enriched from the *Nitrososphaeraceae* family.

Glycosyl transferase family enzymes are predicted to play a crucial role in the production, modification, and/or N-glycosylation of exopolysaccharides (EPS). Additionally, previous studies suggested that the EPS matrix can effectively retain water and nutrients for microbial survival during desiccation (Costa et al., 2018; Roberson et al., 1993). EPS biosynthesis, which is potentially triggered by environmental stresses, offers a selective advantage to the producing microorganisms (Costa et al., 2018; Kumar et al., 2007; Limoli et al., 2015). It is worth pointing out that Kerou et al. (2016) earlier reported that the *Nitrososphaeraceae* family exhibit a higher genetic capability for EPS biosynthesis, implying their potential for adhesion, aggregation, cell-to-cell communication, or biofilm formation. Therefore, it is not surprising that the conserved orthologous clusters linked to biofilm formation are more prevalent in members of the *Nitrososphaeraceae* family compared to other families (Supplementary Table). Similarly, Abiola et al. (2024) reported that *N. viennensis* EN76, a member of the *Nitrososphaeraceae* family, grew as microcolonies on floating filters when observed under a fluorescence microscope. Taken together, a higher capability for EPS biosynthesis might possibly support the growth of the cultivated members of the *Nitrososphaeraceae* family on the floating membrane filter.

Adenylyl cyclases are enzymes responsible for synthesizing cyclic adenosine 3',5'-monophosphate (cAMP), an essential signaling molecule. Consequently, they play a pivotal role in cAMP signaling and regulation pathways (Khannpnavar et al., 2020). The most prevalent class of these enzymes, class III adenylyl cyclases, are frequently associated with domains that enable them to sense and respond to changes in various environmental stimuli (Bassler et al., 2018). Thioredoxin (Trx) plays a vital role in maintaining the redox balance of antioxidant enzymes by enhancing their catalytic activity. This enhancement facilitates the efficient detoxification of reactive oxygen species (ROS) and offers protection against oxidative stress (AlOkda and Van Raamsdonk, 2023; Lu and Holmgren, 2012). Furthermore, Trx interacts with transcription factors that control the expression of genes related to antioxidant defense mechanisms (AlOkda and Van Raamsdonk, 2023). Together, these conserved orthologous clusters within the *Nitrososphaeraceae* family might contribute to their culturability and adaptation to diverse environmental conditions. In the same way, they might also play a role in their growth on the floating membrane filter.

The genus “*Ca*. Nitrosocosmicus” possesses unique core proteins not found in the sister clade, *Nitrososphaera* (Supplementary Table). These include extra copies of adenylate cyclase (class III), TolA protein, sensor histidine kinases, response regulators, NosD-domain containing protein, alcohol dehydrogenase-like family, stress response proteins, cytochrome P450 family, gamma carbonic anhydrase-like family, alpha/beta hydrolase family protein, additional copies of glycosyltransferase family protein (Supplementary Table). Conversely, the genus *Nitrososphaera* features extra copies of proteins associated with DNA repair and recombination mechanisms and transcriptional regulators.

Tol-Pal proteins are essential for maintaining membrane integrity (Lazzaroni et al., 1999; Li et al., 2022; Masilamani et al., 2018) and among these proteins, TolA is particularly important for cell survival and adaptation to diverse stress conditions (Su et al., 2024). Also, sensor histidine kinases (SHKs) are membrane-bound proteins that play a vital role in two-component signal transduction systems (TCSs) (Zschiedrich et al., 2016). These systems typically consist of two paired regulatory proteins: the SHK, which serves as a sensor and transmitter by detecting environmental changes, and the response regulator (RR), which mediates the adaptive response by modifying gene expression (Ma and Phillips-Jones, 2021). TCSs are involved in essential bacterial processes, including regulating bacterial growth, biofilm formation and development, and quorum sensing (Ma and Phillips-Jones, 2021). Possibly, these proteins might be playing a similar role as in bacteria, potentially contributing to the adaptation of members of the genus “*Ca*. Nitrosocosmicus” to diverse soil habitats.

**Fig. 4.**
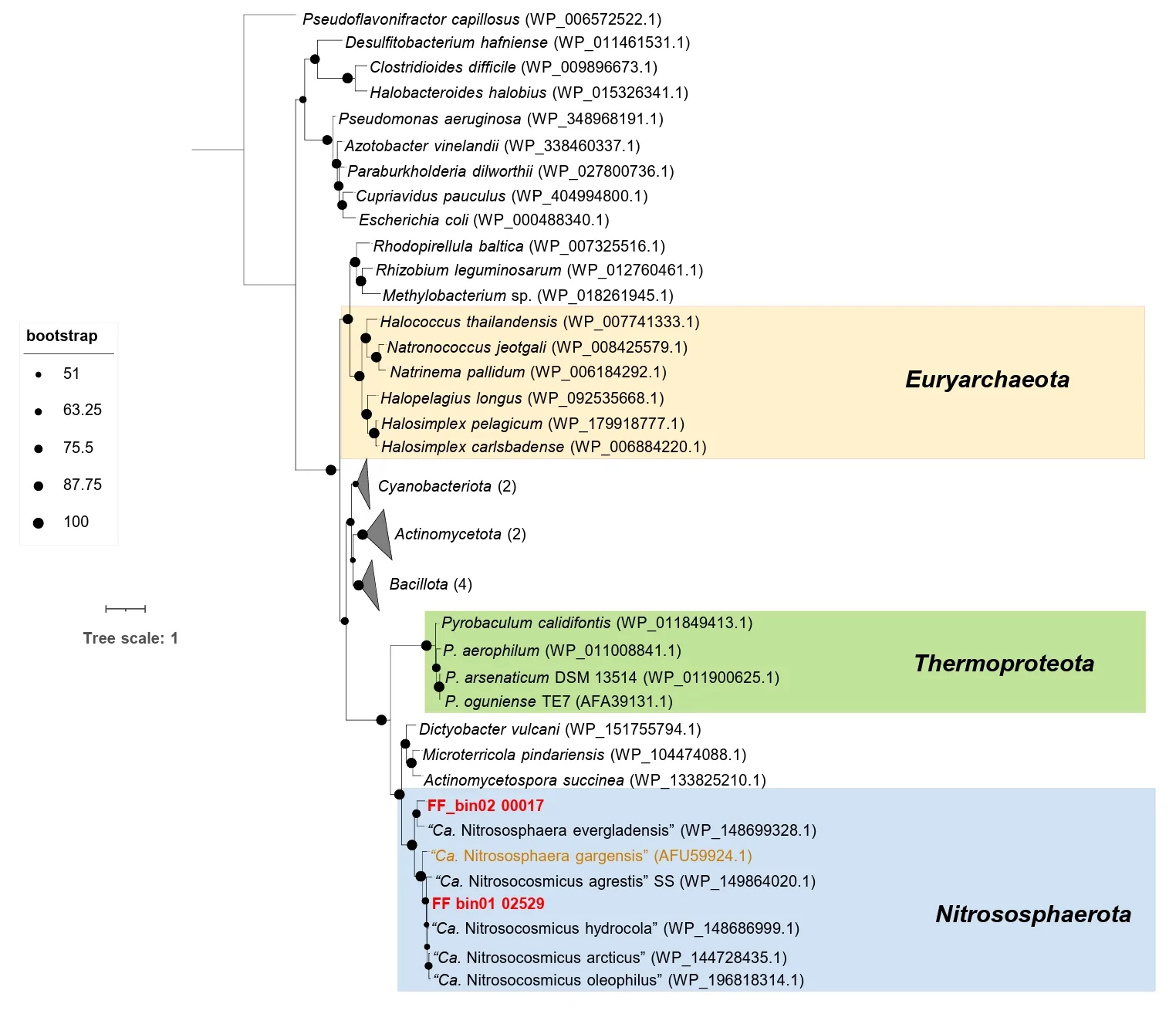
Maximum-likelihood phylogenetic tree of Mn_catalase protein sequences from AOA. The unrooted tree was constructed with IQ-TREE (IQ-TREE options: -B 1000 -m MFP) using aligned Mn_catalase protein sequences from AOA with the inclusion of other bacteria Mn_catalase. The tree was rooted with the outgroup *Pseudoflavonifractor capillosus* (WP_006572522.1), as originally published in Bernroitner et al. (2009) and Zamocky et al. (2011). Bootstrap values based on 1,000 replications are indicated. The scale bar represents 1 change per amino acid position. The two Mn_catalase protein sequences from AOA bins recovered in this study are highlighted in red, while the truncated Mn_catalase containing AOA is marked in brown.

Intriguingly, the AOA bins recovered in this study, along with the strain “*Ca*. Nitrososphaera evergladensis” (Zhalnina et al., 2014) and most of the members of the genus “*Ca*. Nitrosocosmicus”, except “*Ca*. Nitrosocosmicus franklandus”, harbor a gene encoding the manganese catalase (Mn_catalase). Phylogenetic analysis indicates that Mn_catalase found in FF_bin01 and FF_bin02 belong to “*Ca*. Nitrosocosmicus” and *Nitrososphaera*, respectively (Fig. 4). Mn_catalase found in FF_bin01 shares 85.62% and 94.44% identity with “*Ca*. Nitrosocosmicus agrestis” SS and “*Ca*. Nitrosocosmicus hydrocola”, respectively. Similarly, Mn_catalase in FF_bin02 shares 75.20% identity with “*Ca*. Nitrososphaera evergladensis”. Additionally, Mn_catalase appears to have been horizontally acquired by the *Nitrososphaeraceae* family and subsequently lost by some members due to gene loss. However, further genomic and evolutionary analyses are needed to confirm these events.

Interestingly, Abiola et al. (2024) highlighted the differences in ammonia oxidation activity of *N. viennensis* EN76 cells with and without ROS scavengers in the media beneath the filters. The result indicated that the addition of catalase (10 U/ml) and an increased concentration of pyruvate (1 mM) in the media beneath the filter significantly enhanced the ammonia oxidation activity of lower inoculum size of *N. viennensis* EN76 cells on floating filters, which exhibited no activity without these ROS scavengers. It is worth pointing out that *N. viennensis* EN76 has no Mn_catalase gene in its genome. Further, AOA with Mn_catalase were reported to dominate plant rhizospheres with potentially high ROS production (Lee et al., 2024). Together, it is tempting to suggest that the presence of Mn_catalase in our AOA bins might play a role in their growth on the floating membrane filter. Protein families unique to our AOA bins, FF_bin01 (23) and FF_bin02 (12) were annotated as hypothetical proteins (Supplementary Table). This constraint impedes a more thorough investigation of the distinctive characteristics that might have contributed to their growth specifically on the floating membrane filter.

**Fig. 5.**
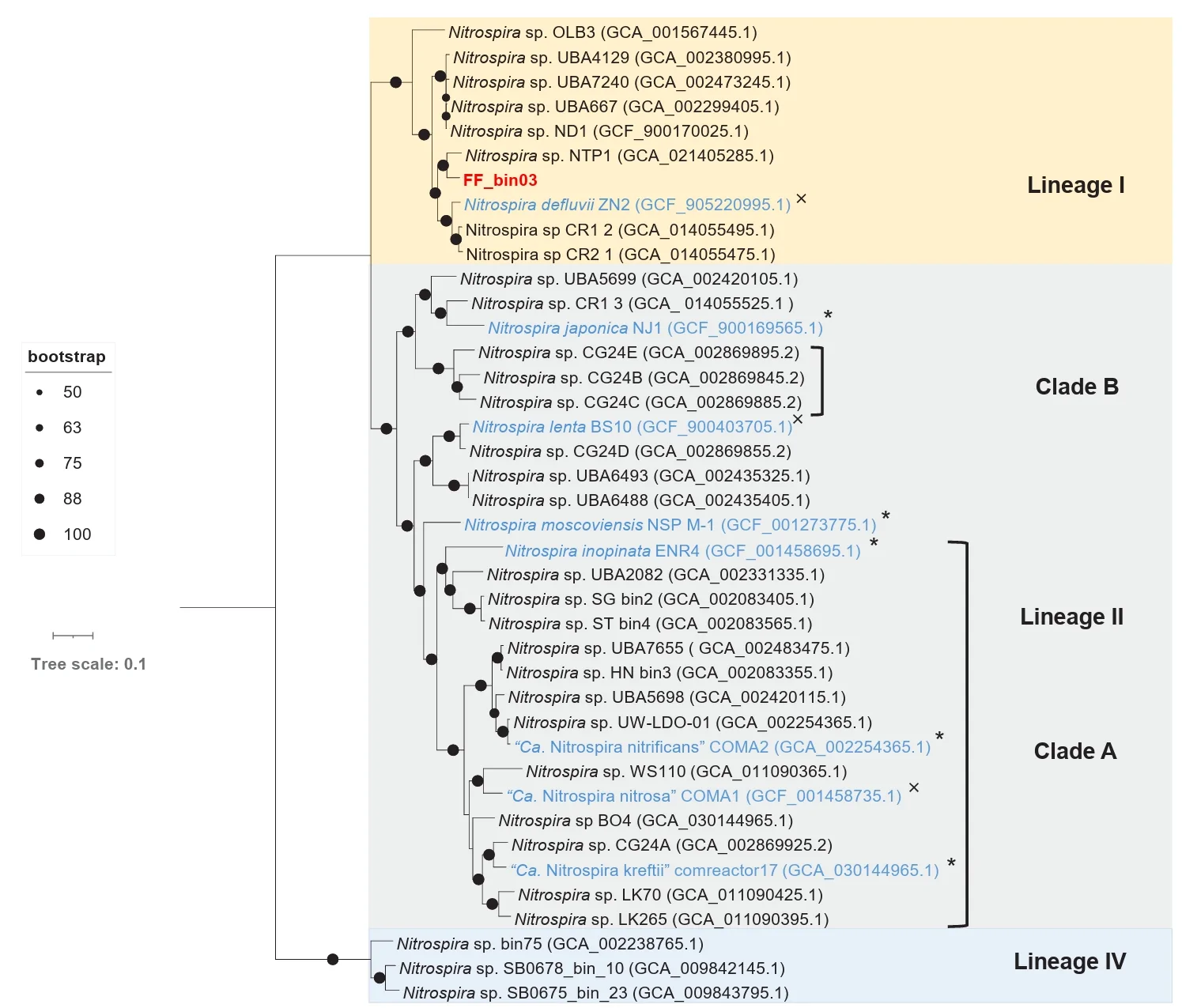
A maximum likelihood phylogenomic tree of the recovered nitrite-oxidizing *Nitrospira* bin (FF_bin03) and reference *Nitrospira* genomes. The tree was inferred based on the concatenated alignment of 71 single-copy core bacterial genes. Bootstrap values based on 1,000 replications are indicated. The scale bar represents a 0.1 change per nucleotide position. *Nitrospira* lineages are indicated by coloured boxes and labeled with roman numerals, comammox clades (A and B) are designated by square brackets. The nitrite-oxidizing bacterium bin (FF_bin03) recovered in this study is highlighted in red, while other isolated/enriched genomes are highlighted in blue. The symbols * and × indicate genomes with or without catalase gene, respectively.

The draft genome of FF_bin03 contains all the key genes required for nitrogen metabolism and assimilation of nitrite-oxidizing *Nitrospira*. Similarly, essential enzymes involved in carbon fixation via the reductive tricarboxylic acid (rTCA) cycle, including all subunits of pyruvate ferredoxin oxidoreductase, fumarate hydratase, and succinate dehydrogenase/fumarate reductase, are found in FF_bin03 genome (Table S2). Furthermore, phylogenomic analysis of FF_bin03 with other *Nitrospira* NOB genomes revealed that it clustered with the canonical *Nitrospira* NOB within Lineage I (Fig. 5). The ANI values between FF_bin03 and the two closest *Nitrospira* genomes, *Nitrospira* sp. NTP1 and *Nitrospira defluvii*, were 84.18% and 79.43%, respectively, which are well below the recommended thresholds for species delineation. Interestingly, similar to *Nitrospira* sp. NTP1, a metagenome-assembled genome (MAG) recovered from the sludge of a bioreactor (Huo et al., 2020), FF_bin03 contains a gene encoding heme catalase. In contrast, *Nitrospira defluvii* from activated sludge enrichment culture lack the catalase gene (Lücker et al., 2010). Together, it is tempting to mention that the presence of the catalase gene in FF_bin03 further supports our hypothesis regarding the importance of ROS detoxification or the activity of ROS scavengers like catalase, which might be a factor to consider when adopting the floating membrane filter cultivation technique.

## Conclusion

The two AOA bins recovered from the floating membrane filter cultures, FF_bin01 and FF_bin02, belong to the genera “*Ca*. Nitrosocosmicus” and *Nitrososphaera*, respectively. The third bin, FF_bin03, is a nitrite-oxidizing bacterium within the genus *Nitrospira*. We propose naming FF_bin01 as “*Candidatus* Nitrosocosmicus superficiei (su. per. fi’ ci. ei. L. gen. n. superficiei, of the surface)” strain FF01 and FF_bin02 as “*Ca*. Nitrososphaera edaphica (e. da’ phi. ca. L. fem. adj. edaphica, of the soil)” strain FF02. Genomic analysis of the soil AOA from the *Nitrososphaeraceae* family revealed unique genomic characteristics that may support adaptation to novel cultivation conditions, including solid surfaces. The floating membrane filter cultivation technique, combined with novel cultivation conditions and metagenomic monitoring systems, offers microbial ecologists new avenues for identifying and characterizing previously uncultivated soil nitrifiers.

## Figures and Tables

**Table 1.** Genomic and growth features of the AOA bins in this study, compared to other cultivated representatives of *Nitrososphaeraceae* family

AOA strains	Status	Growth temp (°C)	Growth pH	Genome size (Mb)	Completeness (%)	Contamination (%)	No of Scaffolds	No of contigs	DNA GC %	Protein coding genes	Reference
**“*Ca.* Nitrosocosmicus” clade**											
FF_bin01	Enriched	30	7.5	2.95	95.15	2.91	11	12	34.28	3,248	This study
“*Ca*. Nitrosocosmicus hydrocola” G61	Enriched	33	8.5	2.99	93.35	10.38	1	1	34.0	3,003	Sauder et al. (2017)
“*Ca.* Nitrosocosmicus franklandus” C13	Enriched	37	7.5	2.8	93.67	6.31	1	1	34.0	2,717	Lehtovirta-Morley et al. (2016)
“*Nitrosocosmicus oleophilus*” MY3	Pure	30	7.5	3.4	94.21	12.6	1	1	34.0	3,252	Jung et al. (2016)
“*Ca.* Nitrosocosmicus agrestis” **SS**	Enriched	37	6.5-7.0	3.2	95.25	10.61	43	44	33.5	3,064	Liu et al. (2021)
“*Ca.* Nitrosocosmicus arcticus” Kfb	Enriched	20	7-7.5	2.6	93.87	9.06	22	27	34.0	2,681	Alves et al. (2019)
***Nitrososphaera* clade**											
FF_bin02	Enriched	30	7.5	1.94	97.09	0.97	3	3	55.79	2,403	This study
*Nitrososphaera viennensis* EN76	Pure	42	7.5	2.5	98.43	5.92	1	1	52.5	2,935	Stieglmeier et al. (2014)
“*Ca.* Nitrososphaera gargensis” Ga9.2	Enriched	46	7.8	2.83	98.87	2.45	1	1	48.5	3,544	Hatzenpichler et al. (2008), Spang et al. (2012)
“*Ca.* Nitrososphaera evergladensis” SR1	Enriched	42	8.0	3.0	97.86	7.26	1	1	50.0	3,302	Zhalnina et al. (2014)

## Data Availability

The whole genome sequences of the AOA bins, “*Ca*. Nitrosocosmicus” strain FF01 and “Ca. Nitrososphaera” strain FF02 recovered in this study have been deposited at the National Center for Biotechnology Information (NCBI) under the BioProject ID: PRJNA1218515 and PRJNA1219171; with the accession JBLNIG000000000 and JBLNIH000000000, respectively.

## Supplementary Information

Table S1. An overview of all the bins recovered in this study

Table S2. Key metabolic features of FF_bin03 recovered from floating membrane filter

Fig. S1. Heatmap result showing the average nucleotide identity (ANI) and Average amino acid identity (AAI). Pairwise ANI (A) and AAI (B) values between the AOA bins, FF_bin01 and FF_bin02, and other AOA reference genomes used to reconstruct the phylogenomic tree in Fig. 2. The two AOA bins recovered in this study are highlighted in red.

Fig. S2. A partition of the pan-genomic matrix of the class Nitrososphaeria. (A) concentric circle showing the core, shell, and cloud orthologous clusters in all the analyzed AOA genomes, (B) a vertical bar chart depicting the number of shell and cloud orthologous clusters in each genome. Shell orthologous clusters were found in at least three of the AOA genomes analyzed, while cloud orthologous clusters were present in not more than two.
