# Supplementary material for: Genomic profiling of soil nitrifying microorganisms enriched on floating membrane filter
Source: J Microbiol. 2025 Apr 29;63(4):e2502002. doi: 10.71150/jm.2502002 (PMC13577086; doi:10.71150/jm.2502002)
Supplement: Supplementary file 1 [file jm-2502002-Supplementary-Fig-S1.pdf]

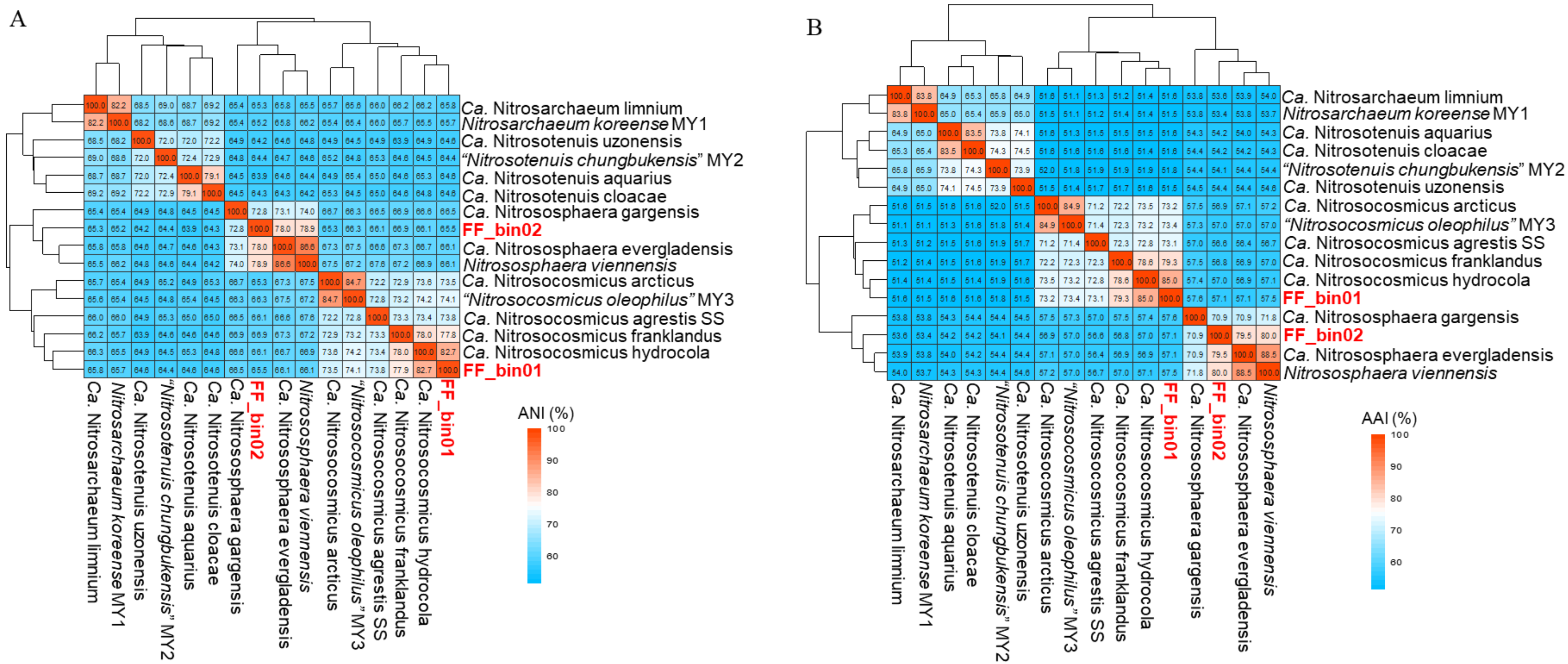

Fig. S1. Heatmap result showing the average nucleotide identity (ANI) and Average amino acid identity (AAI). Pairwise ANI (A) and AAI (B) values between the AOA bins, FF_bin01 and FF_bin02, and other AOA reference genomes used to reconstruct the phylogenomic tree in Fig. 2. The two AOA bins recovered in this study are highlighted in red.
