# Supplementary material for: Genomic profiling of soil nitrifying microorganisms enriched on floating membrane filter
Source: J Microbiol. 2025 Apr 29;63(4):e2502002. doi: 10.71150/jm.2502002 (PMC13577086; doi:10.71150/jm.2502002)
Supplement: Supplementary file 2 [file jm-2502002-Supplementary-Fig-S2.pdf]

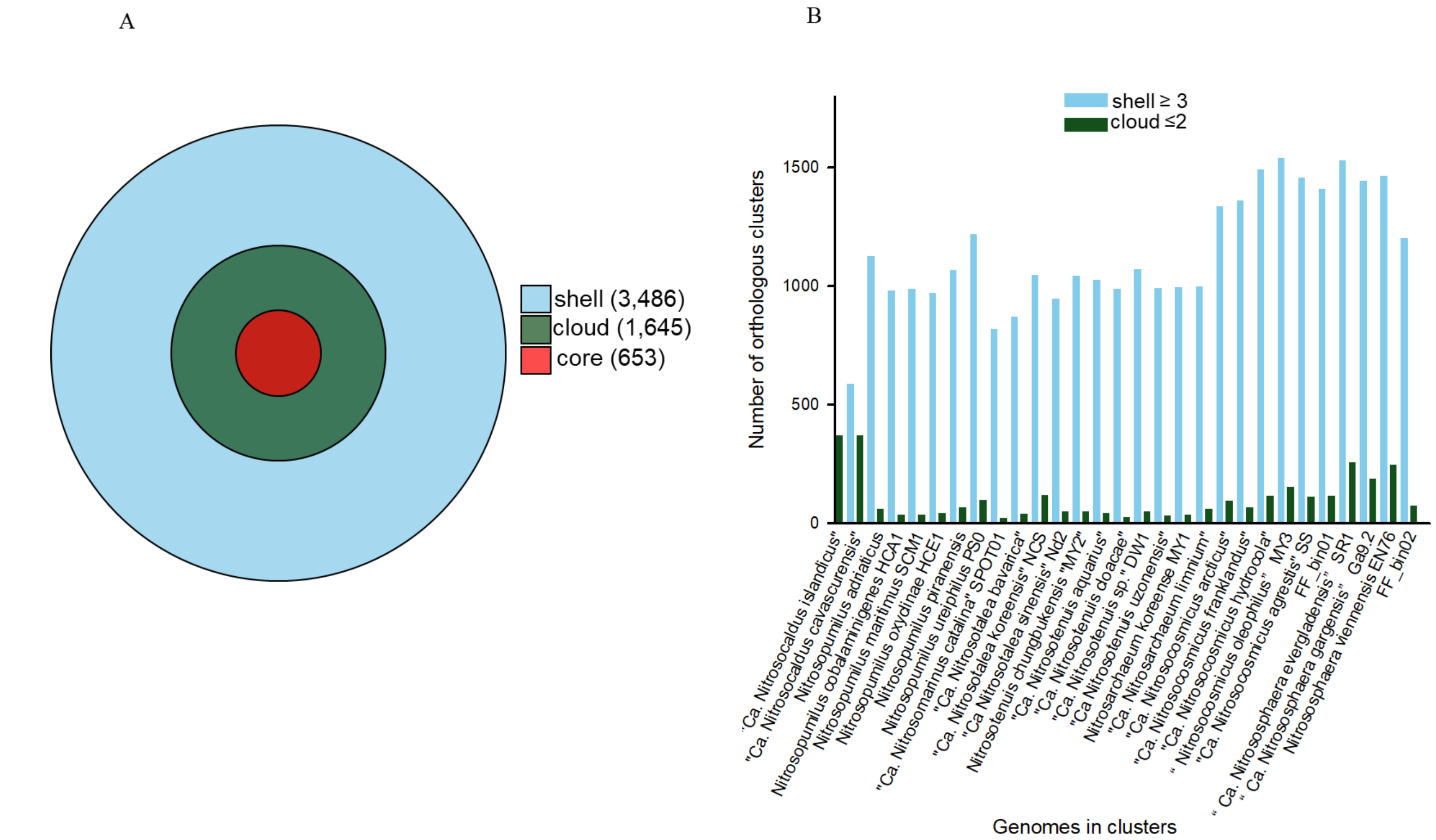

Fig. S2. A partition of the pan-genomic matrix of the class *Nitrososphaeria*. (A) concentric circle showing the core, shell, and cloud orthologous clusters in all the analyzed AOA genomes, (B) a vertical bar chart depicting the number of shell and cloud orthologous clusters in each genome. Shell orthologous clusters were found in at least three of the AOA genomes analyzed, while cloud orthologous clusters were present in not more than two.
