# Supplementary material for: Genomic profiling of soil nitrifying microorganisms enriched on floating membrane filter
Source: J Microbiol. 2025 Apr 29;63(4):e2502002. doi: 10.71150/jm.2502002 (PMC13577086; doi:10.71150/jm.2502002)
Supplement: Supplementary file 3 [file jm-2502002-Supplementary-Table-S1.pdf]

Table S1. An overview of all the bins recovered in this study

**Characteristics**	**FF_bin01**	**FF_bin02**	**FF_bin03**
Lineage	Archaea	Archaea	Bacteria
Scaffold Coverage	119.39×	81.45×	29.1×
Genome Completeness (%)	95.15	97.09	87.67
Contamination (%)	2.91	0.97	14.26
No of scaffolds	11	3	163
No of contigs	12	3	163
N50 (scaffolds)	870539	1168865	48776
N50 (contigs)	772176	1168865	48776
Longest scaffold	1005776	1168865	163477
Longest contig	870539	1168865	163477
DNA GC%	34.28	55.79	57.99
Protein coding genes	3,248	2,403	4,367
Genome size (Mb)	2.95	1.94	4.33
