# Supplementary material for: Genomic profiling of soil nitrifying microorganisms enriched on floating membrane filter
Source: J Microbiol. 2025 Apr 29;63(4):e2502002. doi: 10.71150/jm.2502002 (PMC13577086; doi:10.71150/jm.2502002)
Supplement: Supplementary file 4 [file jm-2502002-Supplementary-Table-S2.pdf]

Table S2. Key metabolic features of FF_bin03 recovered from floating membrane filter

Pathway	Function	Gene name	Gene ID	K0_ID
**Nitrogen metabolism**	Nitrite oxidation	Nitrite/nitrate oxidoreductase, alpha and beta subunit	nxrA and nxrB	K00370 and K00371
	Urea hydrolysis	Urease and Urease accessory proteins	UreABC, UreE, UreDFG,	K21929, K03190, K03189,K03188
	Nitrogen assimilation	Cyanate hydratase	cynS	K01725
		Glutamate dehydrogenase	gdhA	K00261
		Glutamine synthetase	glnA	K01915
		Glutamate synthase	gltB	K00266
**Carbon fixation**	Reductive Tricarboxylic acid (TCA) cycle	pyruvate ferredoxin oxidoreductase alpha subunit	forA	K00169
		pyruvate ferredoxin oxidoreductase gamma subunit	porC	K00172
		Pyruvate:ferredoxin oxidoreductase beta subunit	forB	K00170
		malate dehydrogenase	mdh	K00024
		pyruvate carboxylase subunit B	pycB	K01960
		pyruvate carboxylase subunit A	pycA	K01959
		Fumarate hydratase, class II	fumC	
		succinyl-CoA synthetase alpha subunit	sucD	K01902
		succinyl-CoA synthetase beta subunit	sucC	K01903
		Succinate dehydrogenase/fumarate reductase	sdhC	K00241
		Pyruvate, phosphate dikinase	ppdK	K01006
